# Combination treatment with oncolytic Vaccinia virus and cyclophosphamide results in synergistic antitumor effects in human lung adenocarcinoma bearing mice

**DOI:** 10.1186/1479-5876-12-197

**Published:** 2014-07-17

**Authors:** Elisabeth Hofmann, Stephanie Weibel, Aladar A Szalay

**Affiliations:** 1 Department of Biochemistry, Biocenter, University of Wuerzburg, D-97074 Wuerzburg, Germany; 2 Rudolf Virchow Center, Research Center for Experimental Biomedicine, University of Wuerzburg, D-97078, Wuerzburg, Germany; 3 Institute for Molecular Infection Biology, University of Wuerzburg, D-97078 Wuerzburg, Germany; 4 Department of Radiation Medicine and Applied Sciences and Center for Advanced Radiotherapy Technologies, University of California San Diego, La Jolla, San Diego, CA 92093, USA; 5 Genelux Corporation, San Diego Science Center, 3030 Bunker Hill Streeet, Suite 310, San Diego, CA 92109, USA

**Keywords:** Vaccinia virus, Chemotherapy, Combination therapy, Cyclophosphamide, Lung cancer

## Abstract

**Background:**

The capacity of the recombinant Vaccinia virus GLV-1h68 as a single agent to efficiently treat different human or canine cancers has been shown in several preclinical studies. Currently, its human safety and efficacy are investigated in phase I/II clinical trials. In this study we set out to evaluate the oncolytic activity of GLV-1h68 in the human lung adenocarcinoma cell line PC14PE6-RFP in cell cultures and analyzed the antitumor potency of a combined treatment strategy consisting of GLV-1h68 and cyclophosphamide (CPA) in a mouse model of PC14PE6-RFP lung adenocarcinoma.

**Methods:**

PC14PE6-RFP cells were treated in cell culture with GLV-1h68. Viral replication and cell survival were determined by plaque assays and 3-(4,5-dimethylthiazol-2-yl)-2,5-diphenyltetrazolium bromide (MTT) assays, respectively. Subcutaneously implanted PC14PE6-RFP xenografts were treated by systemic injection of GLV-1h68, CPA or a combination of both. Tumor growth and viral biodistribution were monitored and immune-related antigen profiling of tumor lysates was performed.

**Results:**

GLV-1h68 efficiently infected, replicated in and lysed human PC14PE6-RFP cells in cell cultures. PC14PE6-RFP tumors were efficiently colonized by GLV-1h68 leading to much delayed tumor growth in PC14PE6-RFP tumor-bearing nude mice. Combination treatment with GLV-1h68 and CPA significantly improved the antitumor efficacy of GLV-1h68 and led to an increased viral distribution within the tumors. Pro-inflammatory cytokines and chemokines were distinctly elevated in tumors of GLV-1h68-treated mice. Factors expressed by endothelial cells or present in the blood were decreased after combination treatment. A complete loss in the hemorrhagic phenotype of the PC14PE6-RFP tumors and a decrease in the number of blood vessels after combination treatment could be observed.

**Conclusions:**

CPA and GLV-1h68 have synergistic antitumor effects on PC14PE6-RFP xenografts. We strongly suppose that in the PC14PE6-RFP model the enhanced tumor growth inhibition achieved by combining GLV-1h68 with CPA is due to an effect on the vasculature rather than an immunosuppressive action of CPA. These results provide evidence to support further preclinical studies of combining GLV-1h68 and CPA in other highly angiogenic tumor models. Moreover, data presented here demonstrate that CPA can be combined successfully with GLV-1h68 based oncolytic virus therapy and therefore might be promising as combination therapy in human clinical trials.

## Background

Lung cancer still represents a very fatal disease causing the majority of the cancer-related deaths in males worldwide [1]. Non-small cell lung cancer (NSCLC), including adenocarcinoma, squamous cell carcinoma, and large cell carcinoma, constitutes 80-85% of all lung cancers, whereas small cell lung cancer accounts for 15% to 20%, with most patients having advanced inoperable disease at the time of diagnosis. The current treatment options are surgical resection, platinum-based doublet chemotherapy, and radiation therapy alone or in combination [2]. However, the prognosis for lung cancer patients still remains poor with an overall 5-year survival rate of only 15% [3]. Therefore, new, effective, therapeutic approaches for lung cancer are mandatory. One novel strategy represents the targeted therapy. Therein drugs are applied that specifically target genetic mutations and signalling pathways altered in lung cancer [4]. Up to now four targeted therapies have been FDA-approved for the treatment of lung cancer. Morevoer, the use of oncolytic viruses is a very promising therapeutic approach for the treatment of cancer. These viruses, either naturally occurring or genetically engineered, are replication-competent viruses that are able to selectively infect and destroy cancer cells either after intratumoral or systemic administration [5]. Among the most intensively investigated oncolytic viruses in preclinical or clinical studies are adenoviruses, herpes simplex virus, Newcastle disease virus, measles virus and Vaccinia virus [6-8]. Vaccinia virus (VACV) is a very promising agent for oncolytic virotherapy, since the use of VACV as a vaccine during the eradication of smallpox clearly demonstrated its safety in human patients. Furthermore, the broad host range, the efficient replication exclusively in the cytoplasm of host cells, the natural tropism for tumor tissues, and its large genome with a huge capacity for the integration of recombinant DNA are further advantages over other oncolytic viruses [9,10]. Until now, various oncolytic VACV have been investigated in preclinical and clinical studies [11]. Recently, it was shown that an oncolytic VACV, GLV-1h68, which was constructed by inserting gene expression cassettes for a Renilla luciferase-*Aequora* green fluorescent fusion protein (RUC-GFP), β-galactosidase and β-glucuronidase into the *F14.5 L*, *J2R*, and *A56R* loci, respectively, possesses reduced toxicity and enhanced tumor targeting specificity compared with its parental LIVP strain [12]. We have demonstrated that treatment with GLV-1h68 or its derivatives led to inhibition of tumor growth in several different xenograft models, including human breast cancer [12-14], anaplastic thyroid carcinoma [15], malignant pleural mesothelioma xenografts [16], squamous cell carcinoma [17], pancreatic carcinoma [18-21], prostate carcinoma [22-24], lung carcinoma [24], fibrosarcoma [25], hepatocellular carcinoma [26] as well as canine mammary adenoma [27] and carcinoma [28]. In addition, two clinical trials with GL-ONC1 (clinical grade GLV-1h68) have been successfully started (http://www.clinicaltrials.gov; references NCT00794131 and NCT01443260).

Due to limitiations of oncolytic tumor therapy observed in several clinical studies, various approaches to enhance the efficiency of oncolytic viruses e.g. by combination with different cancer treatment modalities such as chemotherapy or radiotherapy are currently intensively investigated [29]. Improved results have been observed for GLV-1h68 using combinations with cisplatin or gemcitabine in pancreatic [18], mitomycin C in prostate [23] or the β-galactosidase-activatable prodrug seco-analog of duocarmycin SA in breast tumor xenografts [14]. Moreover, systemically delivered GLV-1h68 in combination with focal ionizing radiation (IR) resulted in improved tumor growth inhibition and mouse survival in a glioma tumor model [30].

Cyclophosphamide (CPA) is an alkylating agent that is known to cause crosslinking of DNA and is used for treatment of various tumors. CPA *per se* is an inactive prodrug that requires metabolic activation in the liver to become the active compound 4-hydroxycyclophosphamide. CPA has already been applied in combination with oncolytic viruses in various tumor models and its synergistic effects have been observed with Herpes simplex virus in glioma models [31-34], sarcoma [35] or in lung adenocarcinoma [36], with adenovirus in a hamster model of renal adenocarcinoma [37] and in cancer patients [38], with Reovirus in mouse melanoma [39,40], and with VACV in a glioma xenograft model [41].

In this study, we set out to evaluate the oncolytic activity of GLV-1h68 in the human lung adenocarcinoma cell line PC14PE6 in cell culture, as well as to determine the antitumor potency of GLV-1h68 as monotherapy or in combination with CPA in a mouse model of PC14PE6-RFP lung adenocarcinoma. Our results demonstrate that GLV-1h68 is able to replicate in and kill human PC14PE6-RFP cells in cell culture. Furthermore, GLV-1h68 efficiently colonizes and notably delays the growth of PC14PE6-RFP tumors in a xenograft mouse model. Moreover, combination therapy with CPA and GLV-1h68 significantly improves the antitumoral efficacy of systemically injected GLV-1h68. Higher levels of pro-inflammatory cytokines and chemokines are seen in tumors of GLV-1h68-treated mice, while after CPA or combination treatment factors either expressed by endothelial cells or present in the blood are found to be reduced. Moreover, combination treatment led to a loss of the hemorrhagic phenotype of PC14PE6-RFP tumors. Our results strongly suggest that the enhanced tumor control achieved by combining GLV-1h68 with CPA is due to an action of CPA on the tumor vasculature.

## Methods

### Cell lines and virus strain

Stably dsRed2-expressing human PC14PE6 cells were engineered and kindly provided by the group of F. Winkler (University of Heidelberg, Neurooncology, Heidelberg, Germany) in 2008 [42]. This PC14PE6-RFP cells were authenticated by the Leibniz-Institut DSMZ (Deutsche Sammlung von Mikroorganismen und Zellkulturen GmbH, Braunschweig, Germany) to be identical with the parental cell line PC14 (Riken, Japan) in 2012. PC14PE6-RFP cells were maintained in DMEM (PAA Laboratories, Cölbe, Germany) supplemented with 10% fetal bovine serum (FBS), 2 mM GlutaMAX (both from Invitrogen GmbH, Karlsruhe, Germany), 1× non-essential amino acids, 1× penicillin/streptomycin (both from PAA Laboratories) at 37°C under 5% CO_2_. African green monkey kidney fibroblasts (CV-1; ATCC number CCL-70) were cultured in growth medium consisting of DMEM with 10% FBS and 1x penicillin/streptomycin at 37°C under 5% CO_2_. The attenuated Vaccinia virus strain GLV-1h68 was derived from LIVP (Lister strain from the Institute of Viral preparations, Moscow, Russia), as described previously [12].

### Viral replication assay

For the viral replication assay, PC14PE6-RFP cells grown in 24-well plates were infected with GLV-1h68 at MOI 0.1 or 1.0 in infection medium (DMEM containing 2% FBS and supplements). After one hour of incubation at 37°C with gentle agitation every 20 min, virus-containing supernatants were removed and replaced by fresh growth medium. After 24, 48 or 72 h, cells and supernatants were harvested. Following three freeze-thaw cycles, serial dilutions of the lysates were titered by standard plaque assays on CV-1 cells. All samples were measured in triplicate.

### Cell viability assay

To determine viral cytotoxicity, GLV-1h68 infected and uninfected PC14PE6-RFP cells in 24-well plates were analyzed using 3-(4,5-dimethylthiazol-2-yl)-2,5-diphenyltetrazolium bromide (MTT) assay. Cells were infected with GLV-1h68 at MOI 0.1 or 1.0 or mock-infected with infection medium (DMEM containing 2% FBS and supplements). After one hour of incubation, virus-containing supernatants were removed by aspiration and fresh medium was added. After 24, 48 and 72 h, respectively, media were removed and 500 μl MTT (2.5 mg/ml, Sigma-Aldrich, Germany) solution in DMEM without phenol red (PAA Laboratories, Cölbe, Germany) was added for 2 h at 37°C and 5% CO_2_. MTT solution was then removed and 400 μl of 1 N HCl in isopropanol was added. Each sample (3×100 μl) was transferred to a 96-well plate and absorbance was measured at 570 nm with a reference wavelength of 650 nm in a Sunrise Microplate reader (Tecan, Austria). The percentage of cell survival was calculated using the following formula: % cell survival = (absorbance value of infected cells/absorbance value of uninfected control cells) × 100%.

### Human tumor xenografts and virus injections

All animal experiments were performed in accordance with protocols approved by the government of Unterfranken (Wuerzburg, Germany, protocol number AZ 55.2-2531.01-17/08) or by the Institutional Animal Care and Use Committee (IACUC) of Explora Biolabs (San Diego, USA, protocol number EB11-025). Human PC14PE6-RFP cells (4×10^5^ cells in 100 μl PBS) were implanted subcutaneously into the right flank of six-week-old female nude mice (Hsd/Athymic Nude-Foxn1^nu^, Harlan Laboratories, Netherlands and Indianapolis). Tumor growth was monitored twice a week using a digital caliper. Tumor volume was calculated with the following formula: [(length × width^2^) × 0.52]. When tumors reached 100–200 mm^3^ mice were either injected via the tail vein with 1×10^7^ pfu GLV-1h68 (in 100 μl PBS) or PBS as control and/or combination treatment was started (day 0). For cyclophosphamide (CPA) combination treatment groups of infected or uninfected mice were injected intraperitoneally with 140 mg/kg bodyweight CPA (Sigma-Aldrich, Germany) at day 0, and with 100 mg/kg bodyweight CPA at days 1, 3, 7, 10, 15, 18 and 21.

### Virus titration from tumors and organs

To assess viral distribution, PC14PE6-RFP tumor-bearing mice were either infected via tail vein with 1×10^7^ pfu of GLV-1h68 (in 100 μl PBS) or subjected to combination treatment. At indicated time points, mice were sacrificed and tumors and organs were prepared and weighted. Tumors were homogenized in an gentleMACS Dissociator using M-Tubes (both Miltenyi Biotech GmbH, Bergisch Gladbach, Germany) and organs in Precellys tubes (Peqlab, Erlangen, Germany) in a FastPrep™ FP120 (Thermo Electron Corporation, Langenselbold, Germany). After three freeze-thaw cycles, viral titers in homogenates were determined by standard plaque assays on CV-1 cells.

### Fluorescence live-animal imaging

Tumor cell growth and viral infection were monitored directly by optical imaging based on dsRed-expression by tumor cells and GFP-expression by virus infected cells respectively and quantified using a Maestro EX imaging system (CRI, Woburn, MA). Mice were anesthetized by isofluran inhalation (induction 4%, maintenance 1%). Images were taken at days 14 and 21 with a Maestro EX imaging system (CRi, Woburn, MA) using appropriate filters for dsRed (tumor, excitation: 503–555 nm, emission: 580 nm cut-in) and GFP (virus, excitation: 445–490 nm, emission: 515 nm cut-in). Images were evaluated and quantified using the Maestro Version 2.10.0 software.

### Rodent multi-analyte profile

For preparation of tumor lysates, three mice of each group were sacrificed 7 dpi. Tumors were removed, resuspended in 9 volumes (W/V) lysis buffer (50 mM Tris–HCl (pH 7.4); 2 mM EDTA (pH 7.4), 2 mM PMSF and Complete Mini protease inhibitors (Roche, Mannheim, Germany) and lysed in an gentleMACS Dissociator using M-Tubes (both Miltenyi Biotech GmbH, Bergisch Gladbach, Germany). Samples were then centrifuged at 500 g at 4°C for 5 min and supernatants were submitted to Rules-Based Medicine (Myriad RBM, Austin, USA) for bead-based immunodetection of mouse immune-related protein antigens (RodentMAP® v2.0).

### Immunohistochemistry

For immune-histochemistry, tumors were excised and snap-frozen in liquid N_2_, followed by fixation in 4% paraformaldehyde/PBS pH 7.4 for 16 h at 4°C. Fixed tumors were dehydrated in 10% Sucrose/PBS for 3–4 h followed by 30% sucrose/PBS for 12 h and finally embedded in Tissue-Tek® O.C.T. (Sakura Finetek Europe B.V., Alphen aan den Rijn, Netherlands). Tumor samples were sectioned (15 μm) with the cryostat 2800 Frigocut (Leica Microsystems GmbH, Wetzlar, Germany) and stored at -80°C. Antibody-labeling was performed following fixation in ice-cold aceton. Endothelial cells were labelled with the hamster anti-mouse CD31 antibody (Chemicon, International, Temecula, CA) and Cy3-conjugated secondary donkey anti-hamster antibody obtained from Jackson ImmunoResearch (West Grove, PA). The primary antibody was incubated for 1 h. After washing with PBS, sections were labeled for 30 min with the secondary antibody and finally mounted in Mowiol 4–88.

### Fluorescence microscopy

The fluorescence-labelled preparations were examined using the Leica TCS SP2 AOBS confocal laser microscope equipped with an argon, helium-neon and UV laser and the LCS 2.16 software (1024 × 1024 pixel RGB-color images). Digital images were processed with Photoshop 7.0 (Adobe Systems, Mountain View, CA) and merged to yield overlay images.

### Measurements of microvessel density

The vascular density was determined in microscopic images (×20 objective, ×10 ocular) of CD31-labelled tumor sections. On the confocal microscope, the CD31 fluorescence was set to a clearly detectable level by adjusting the photo-multiplier before the images were captured. All images were decorated with five horizontal lines at identical positions using Photoshop 7.0 and all vessels which intersected these lines were counted to yield the vascular density. The vascular density was calculated in duplicate for ten images (five images of two different control, GLV-1h68-infected or combination-treated tumors) and presented as mean values with standard deviations.

### Statistics

A two-tailed Student’s t test was used for statistical analysis. P values of < 0.05 were considered statistically significant.

## Results

### Human lung adenocarcinoma PC14PE6-RFP cells are permissive to infection with vaccinia virus GLV-1h68

**Figure 1:**
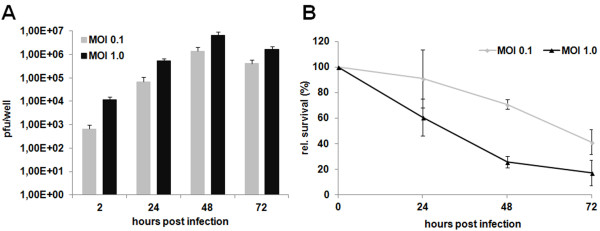
**GLV-1h68 replicates in and kills human PC14PE6-RFP cells in cell culture. (A)** Virus titers were determined from PC14PE6-RFP cells infected with GLV-1h68 at indicated MOIs. **(B)** MTT assays were performed on PC14PE6-RFP cells infected with GLV-1h68 with indicated MOIs. Data are presented as percentage of control cells +/- s.d.

In order to determine the infectivity of GLV-1h68 for the human lung adenocarcinoma cell line PC14PE6, we examined the kinetics of the GLV-1h68 replication in this cell line. Accordingly, PC14PE6-RFP cells were infected with GLV-1h68 (multiplicity of infection (MOI) 0.1 or 1.0), and a time-course analysis of viral replication was performed. A 2- to 3-fold logarithmic increase of GLV-1h68 titers in PC14PE6-RFP cells within 72 hours post infection (hpi) was observed at both MOIs (Figure 1A), demonstrating that the oncolytic Vaccinia virus GLV-1h68 is able to efficiently replicate within the human lung adenocarcinoma cells in cell culture. Next, we analyzed the oncolytic efficacy of GLV-1h68 on PC14PE6-RFP cells by a MTT cell viability assay. The results revealed that 24 hpi the number of surviving cells markedly decreased in an MOI-dependent manner. At 72 hpi, only 41+/-9.7% or 17+/-10.2% of cells survived the treatment at MOI 0.1 or 1.0, respectively, demonstrating the considerable cytotoxic effect of GLV-1h68 on PC14PE6-RFP cells. Taken together, these data indicate that GLV-1h68 productively infects, replicates in and lyses human PC14PE6-RFP cells in cell culture, suggesting that GLV-1h68 has the potential to treat PC14PE6-RFP tumors in a mouse model.

### GLV-1h68 efficiently colonizes PC14PE6-RFP tumors in a xenograft mouse model

Next, we investigated the biodistribution of systemically injected GLV-1h68 in PC14PE6-RFP tumor-bearing nude mice. For that, homogenates of tumors and organs from intravenously injected (1x10^7^ plaque-forming units (pfu)) PC14PE6-RFP tumor-bearing mice (n = 4) were titered on CV-1 cells three, seven and 14 days after injection (Table 1). GLV-1h68 localized primarily to the tumors (in 4/4 mice per group) and was either not detectable (in liver, spleen) or found at notably lower titers in normal tissues (either in 1/4 or 2/4 mice, in lungs, ovaries or brain). Within three days post infection 3.5×10^7^+/-3.15×10^6^ pfu GLV-1h68/g tissue were found in tumors. The highest viral load in tumors was detected 14 days post infection, on average 3.44×10^8^+/-1.85×10^7^ pfu/g tumor tissue. These data suggest that GLV-1h68 has a strong selectivity towards PC14PE6-RFP tumors in nude mice.

**Table 1:** Distribution of GLV-1h68 virus particles in tissues of PC14PE6-RFP tumor-bearing nude mice

**Distribution of GLV-1h68 in tissues of PC14PE6-RFP tumor-bearing nude mice**			
**Tissue**	**pfu/g of tissue (n = 4)**		
	**3 dpi**	**7 dpi**	**14 dpi**
Tumor	3.5x10^7^+/-3.15x10^6^*(4/4)*	1.91×10^8^+/-3.21×10^6^*(4/4)*	3.44×10^8^+/-1.85×10^7^*(4/4)*
Liver	0	0	0
Spleen	0	0	0
Lungs	0	2.14×10^3^+/-8.04×10^2^*(1/4)*	5.04×10^1^+/-1.22×10^1^*(2/4)*
Ovaries	0	3.4×10^3^+/-2.04×10^3^*(1/4)*	9.61×10^3^+/-1.86×10^4^*(2/4)*
Brain	0	0	1.96×10^2^+/-1.58×10^2^*(1/4)*

PC14PE6-RFP tumor-bearing nude mice (n = 4) were infected with 1×10^7^ pfu of GLV-1h68. Three, seven and 14 days after infection, tumors and organs were harvested and viral titers were determined by standard plaque assays on CV-1 cells. Results are shown as mean pfu/g tissue or organ +/- s.d.

### GLV-1h68 treatment significantly delays tumor growth in PC14PE6-RFP bearing nude mice

**Figure 2:**
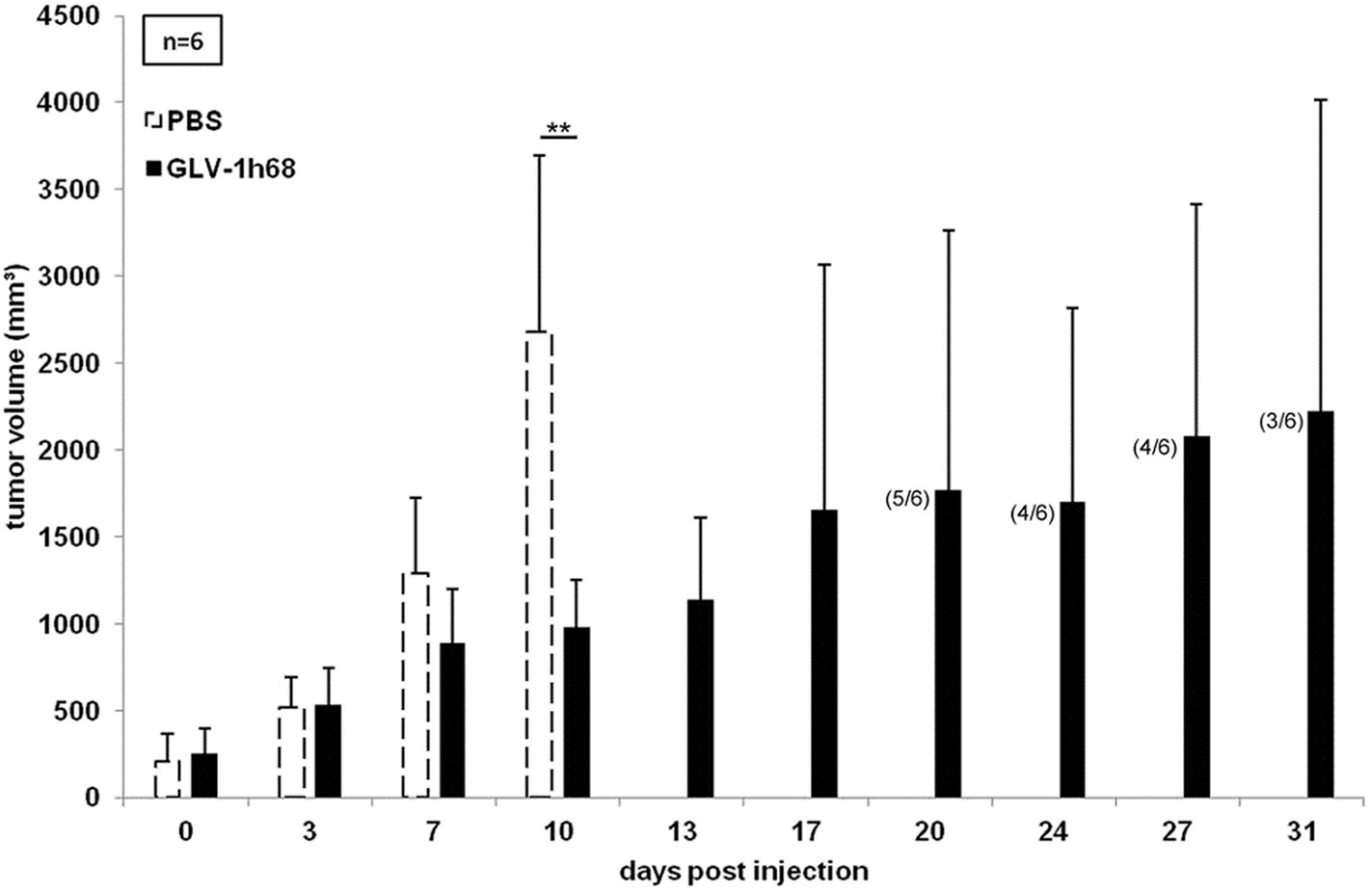
**Treatment of PC14PE6-RFP xenografts by a single i.v. injection of GLV-1h68.** 4×10^5^ PC14PE6-RFP cells were implanted subcutaneously into the right flank of female nude mice. When tumor volumes reached approximately 200–250 mm^3^ mice were randomized into two groups (n = 6). Mice were either treated with an i.v. injection of 1×10^7^ pfu GLV-1h68 or received PBS as control (day 0). Numbers of surviving mice are indicated in parentheses.

Due to the observed oncolytic effect of GLV-1h68 on PC14PE6-RFP cells in cell culture and the massive colonization of PC14PE6-RFP tumors with GLV-1h68, we analyzed the PC14PE6-RFP tumor growth response to oncolytic virotherapy with GLV-1h68 *in vivo*. For that, 4×10^5^ PC14PE6-RFP cells were implanted s.c. into the right flank of nude mice. Tumor-bearing mice (n = 6) were i.v. injected with 1×10^7^ pfu GLV-1h68 or with PBS as control. Tumor growth was monitored and already ten days post injection a significant tumor growth delay (p < 0.01) of the GLV-1h68 treated group compared to the PBS group was observed (Figure 2). After this time point all mice of the control group had to be sacrificed due to high tumor burden. Despite an initial response to therapy, in the course of the experiment tumors of GLV-1h68-treated mice showed steadily tumor growth. Moreover, starting with day 20, individual mice of the GLV-1h68 treated group had to be taken out of the experiment due to excess tumor volume.

### Cyclophosphamide significantly improves the antitumoral efficacy of systemically injected GLV-1h68

**Figure 3:**
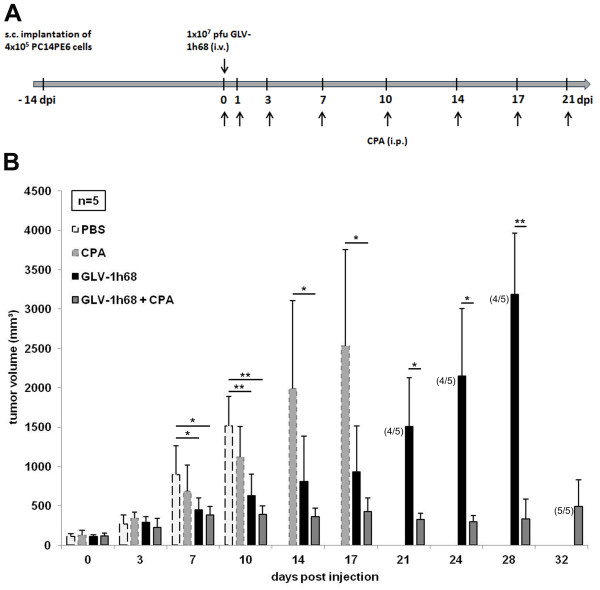
**Combination treatment of PC14PE6-RFP tumor-bearing nude mice with GLV-1h68 and cyclophosphamide. (A)** Treatment schedule demonstrating the regimen of GLV-1h68 and CPA treatment in PC14PE6-RFP tumor-bearing mice**. (B)** Tumor volumes of PC14PE6-RFP tumors treated with or without combination treatment. 4×10^5^ PC14PE6-RFP cells were implanted subcutaneously into the right flank of female nude mice. When tumor volumes reached approximately 100–130 mm^3^ mice were randomized into four groups (n = 5). Mice were either treated with a single i.v. injection of 1×10^7^ pfu GLV-1h68, with multiple i.p. injections of CPA, a combination of both or with PBS as control. Numbers of surviving mice are indicated in parentheses.

Since we have seen that treatment with GLV-1h68 leads to a significant tumor growth delay in the PC14PE6-RFP xenograft model but unfortunately neither tumor regression nor stable disease could be achieved by this therapy, we set out to improve the outcome of the therapy by combining oncolytic virotherapy with chemotherapy using cyclophosphamide (CPA). For that, nude mice bearing subcutaneously implanted PC14PE6-RFP tumors either received a single i.v. injection of 1×10^7^ pfu GLV-1h68 or an i.p. injection of 140 mg/kg/bw CPA at day 0, followed by multiple i.p. injections of 100 mg/kg/bw every 3–4 days during the whole experiment or a combination of viro- and chemotherapy (n = 5; Figure 3A). In PBS treated control mice rapid tumor growth was observed, reaching the tumor end point between ten and 14 days post treatment initiation (Figure 3B). Therapy with CPA alone resulted in a slight delay of tumor growth compared to control mice. Treatment with oncolytic GLV-1h68 resulted in a more pronounced tumor growth retardation being significant from day seven post injection on (p < 0.05, day 7; p < 0.01, day 10). Mice of this group reached highest acceptable tumor volumes on day 28 after start of therapy. In marked contrast, combination treatment showed an impressive antitumor activity. The tumor growth retardation after combination treatment was significant compared to the PBS control group starting day seven (p < 0.05, day 7; p < 0.01, day 10) and compared to CPA alone from 14 days post injection on (p < 0.05, days 14 and 17). Even more, beginning with day 21 combination treatment had a significant better effect on the growth of PC14PE6-RFP tumors than therapy with GLV-1h68 alone (p < 0.05, days 21 and 24; p < 0.01, day 28) with stable tumor volume until 32 days. Over the course of the experiment no evident toxicity by either treatment was seen, as confirmed by monitoring of body weight (data not shown). These results demonstrate that CPA in combination with GLV-1h68 acts synergistically in PC14PE6-RFP xenografts and is promising for future clinical applications.

### Treatment with CPA does not alter viral titers in tumors and organs of nude mice

To address whether the increased antitumor efficacy of the combination therapy is associated with higher viral titers in the tumors or CPA exerts an immunosuppressive effect in our experimental model possibly resulting in elevated viral load in organs, we prepared tumors and organs of GLV-1h68-treated as well as GLV-1h68- and CPA-treated mice 21 days post infection and determined titers by plaque assays. Interestingly, combination treatment does not significantly alter the amounts of viral particles in the tumor compared to treatment with GLV-1h68 alone (Table 2). Furthermore, we did not see increased viral titers in the organs of mice after combination treatment, suggesting that in our experimental model treatment with CPA has no immunosuppressive effect leading to systemic infection with Vaccinia virus.

**Table 2:** Distribution of GLV-1h68 virus particles in tissues of PC14PE6-RFP tumor-bearing nude mice treated with GLV-1h68 or GLV-1h68/CPA

**Distribution of GLV-1h68 in tissues of PC14PE6-RFP tumor-bearing nude mice, 21 dpi**		
**Tissue**	**pfu/g of tissue (n = 3)**	
	**GLV-1h68-treated mice**	**GLV-1h68- and CPA-treated mice**
Tumor	6.35×10^8^+/-9.01×10^7^*(3/3)*	1.42×10^8^+/-5.1×10^6^*(3/3)*
Liver	0	0
Spleen	0	0
Lungs	2.63×10^2^+/-9.43 *(1/3)*	0
Ovaries	1.51×10^4^+/-3.06×10^2^*(2/3)*	7.28×10^4^+/-1.15×10^3^*(1/3)*
Brain	0	0

PC14PE6-RFP tumor-bearing nude mice (n = 3) were either infected solely with 1×10^7^ pfu of GLV-1h68 or infected with GLV-1h68 and received simultaneous treatment with CPA. 21 days after infection, tumors and organs were harvested and viral titers were determined by standard plaque assays on CV-1 cells. Results are shown as mean pfu/g tissue or organ +/- s.d.

### CPA treatment results in infection of larger tumor areas

**Figure 4:**
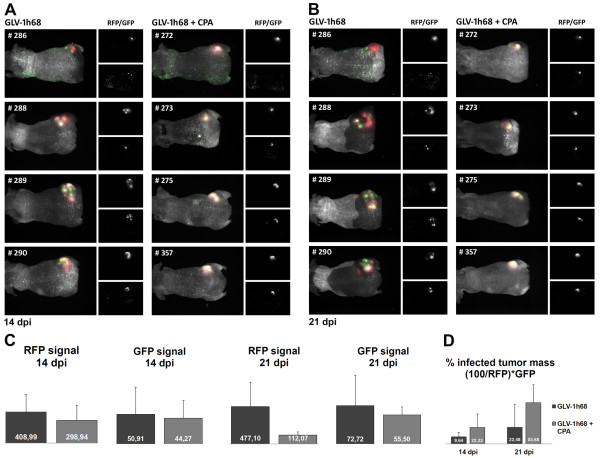
**Imaging of live animals.** Mice were imaged at 14 **(A)** or 21 **(B)** days post initiation of treatment. The images show the tumor area of four representative mice of each group. At the left side of each unit an overlay of white light images of the mice and fluorescence images (RFP: tumor; GFP: infection) are shown. On the top right side separate RFP signals respectively on the bottom right GFP signals are displayed. **(C)** Quantification of tumor burden (RFP signals) and viral infection (GFP signals) by scaled counts/s. **(D)** Calculation of the percent of infected tumor area.

To analyze the intratumoral viral distribution as well as the extent of the viral tumor infection, we investigated live tumor-bearing mice 14 and 21 days after beginning of treatment by fluorescence imaging (Figure 4A and B). For this, the tumor mass was measured via the RFP fluorescence of the tumor cells and the viral infection was determined by GFP expression. The RFP signals obtained by fluorescence imaging (Figure 4C) correlated well with tumor size measurements shown in Figure 3. Twenty-one days post infection GFP signals in GLV-1h68-treated mice were similar to the combination group confirming the results found by plaque assays of homogenized tumors (Table 2). Finally, the percentage of infected tumor mass was calculated (Figure 4D). Strikingly, combination treatment leads to a higher percentage of the tumor mass infected (55.68%) compared to monotherapy with GLV-1h68 (22.48%) 21 dpi. Thus, simultaneous treatment with CPA led to a more pronounced viral distribution within the tumor tissue and, therefore, contingently, to a better oncolytic effect in PC14PE6-RFP tumors.

### Analysis of the host immune response upon treatment with virus and/or CPA

Since CPA is known as a broad immunosuppressive agent, we investigated the host immune response to virus infection and/or CPA treatment 7dpi in PC14PE6-RFP tumor-bearing mice. We are aware of the fact that nude mice lack adaptive immunity and results obtained by these experiments might not be directly transferred to immunocompetent humans. Nevertheless, we used the nude mouse model since we wanted to study the oncolytic effect of Vaccinia virus on a lung adenocarcinoma of human origin. By protein profiling we found that expression levels of several cytokines and chemokines were significantly elevated in tumors of GLV-1h68-treated mice compared to untreated controls (Table 3A). The majority of these cytokines and chemokines, like eotaxin, IL-7, MIP-1 beta, MCP-1, MCP-3, MCP-5, or TNF-alpha, and even Myeloperoxidase secreted by neutrophils and tissue macrophages, create a pro-inflammatory microenvironment in the target tissues. The only protein that was found to be down-regulated upon viral infection was EGF, a growth factor stimulating the growth of epidermal or epithelial tissues and some fibroblasts.

**Table 3:** Comparison of mouse immune-related protein profiling in PC14PE6-RFP-derived tumors at day seven after treatment begin (n = 3)

***Antigen names***	***Ratios***^***a***^	***Classification***
**(A) GLV-1h68/PBS**		
Eotaxin	9.34*	Proinflammatory chemokine
Interleukin-11 (IL-11)	1.90*	Cytokine
Interleukin-2 (IL-2)	1.97*	Cytokine
Interleukin-7 (IL-7)	1.63*	Proinflammatory cytokine
Macrophage Inflammatory Protein-1 beta (MIP-1 beta)	3.76*	Proinflammatory chemokine
Monocyte Chemotactic Protein 1 (MCP-1)	18.30**	Proinflammatory chemokine
Monocyte Chemotactic Protein 3 (MCP-3)	11.12**	Proinflammatory chemokine
Monocyte Chemotactic Protein-5 (MCP-5)	27.85*	Proinflammatory chemokine
Myeloperoxidase (MPO)	3.99**	Peroxidase enzyme
Oncostatin-M (OSM)	1.99*	Cytokine
Tumor Necrosis Factor alpha (TNF-alpha)	2.05*	Proinflammatory cytokine
Epidermal Growth Factor Mouse (EGF Mouse)	0.54*	Growth factor
**(B) GLV-1h68 + CPA/ PBS**		
Macrophage Colony-Stimulating Factor-1 (M-CSF-1)	1.69*	Proinflammatory cytokine
Monocyte Chemotactic Protein 1 (MCP-1)	20.16*	Proinflammatory chemokine
Monocyte Chemotactic Protein-5 (MCP-5)	30.60*	Proinflammatory chemokine
Apolipoprotein A-I (Apo A-I)	0.17*	Anti-inflammatory protein
Epidermal Growth Factor Mouse (EGF Mouse)	0.54*	Growth factor
Fibrinogen	0.52*	Blood coagulation
Vascular Cell Adhesion Molecule-1 (VCAM-1)	0.45*	Cell-cell adhesion
von Willebrand factor (vWF)	0.39*	Blood coagulation
**(C) GLV-1h68/CPA**		
Eotaxin	12.43*	Proinflammatory chemokine
Granulocyte Chemotactic Protein-2 Mouse (GCP-2 Mouse)	2.51*	Proinflammatory chemokine
Growth-Regulated Alpha Protein (KC/GRO)	2.96*	Proinflammatory chemokine
Interleukin-10 (IL-10)	2.74*	Cytokine
Interleukin-12 Subunit p70 (IL-12p70)	2.11*	Pleiotropic cytokine
Macrophage Inflammatory Protein-1 beta (MIP-1 beta)	3.06*	Proinflammatory chemokine
Macrophage Inflammatory Protein-3 beta (MIP-3 beta)	2.90**	Proinflammatory chemokine
Matrix Metalloproteinase-9 (MMP-9)	2.30*	Enzyme
Monocyte Chemotactic Protein 1 (MCP-1)	9.76**	Proinflammatory chemokine
Monocyte Chemotactic Protein 3 (MCP-3)	5.90**	Proinflammatory chemokine
Monocyte Chemotactic Protein-5 (MCP-5)	20.97*	Proinflammatory chemokine
**(D) GLV-1h68 + CPA/CPA**		
Granulocyte Chemotactic Protein-2 Mouse (GCP-2 Mouse)	3.05*	Proinflammatory chemokine
Interleukin-6 (IL-6)	5.01*	Cytokine
Monocyte Chemotactic Protein 1 (MCP-1)	10.75*	Proinflammatory chemokine
Monocyte Chemotactic Protein-5 (MCP-5)	23.04*	Proinflammatory chemokine
Serum Amyloid P-Component (SAP)	1.38*	Acute phase reactant
T-Cell-Specific Protein RANTES (RANTES)	3.32*	Proinflammatory chemokine
Tissue Inhibitor of Metalloproteinases 1 Mouse (TIMP-1 Mouse)	2.56*	Protein inhibitor
Vascular Cell Adhesion Molecule-1 (VCAM-1)	0.78*	Cell-cell adhesion
**(E) CPA/PBS**		
von Willebrand factor (vWF)	0.48*	Blood coagulation
**(F) GLV-1h68 + CPA/GLV-1h68**		
von Willebrand factor (vWF)	0.55**	Blood coagulation

Note: All ratios greater than 1 indicate an up-regulation of protein expression, and all ratios less than 1 indicate down-regulation. ^a^Significant modification in protein ratios with *p < 0.05, **p < 0.01, ***p < 0.001, Student’s t -test.

Combination treatment leads to significant up-regulation of M-CSF-1, MCP-1 and MCP-5 (pro-inflammatory) and down-regulation of Apo A-I (anti-inflammatory) and EGF (Table 3B) compared to untreated controls. Interestingly, levels of VCAM-1 (expressed on endothelial cells during inflammation) and coagulation factors Fibrinogen and vWF are also significantly decreased after combination treatment.

Compared to CPA treatment alone, infection with GLV-1h68 or combination treatment leads to up-regulation of several pro-inflammatory cytokines and chemokines (Table 3C and D), like seen in the virus-treated versus untreated tumors, affirming that virus infection results in a pro-inflammatory status within the tumor. Moreover, compared to monotherapy with CPA, VCAM-1 is decreased upon combination treatment. Interestingly, after CPA or combination therapy lower levels of the von Willebrand factor (vWF) are found (Table 3B, E and F).

### The hemorrhagic phenotype of PC14PE6-RFP tumors disappears after combination treatment

**Figure 5:**
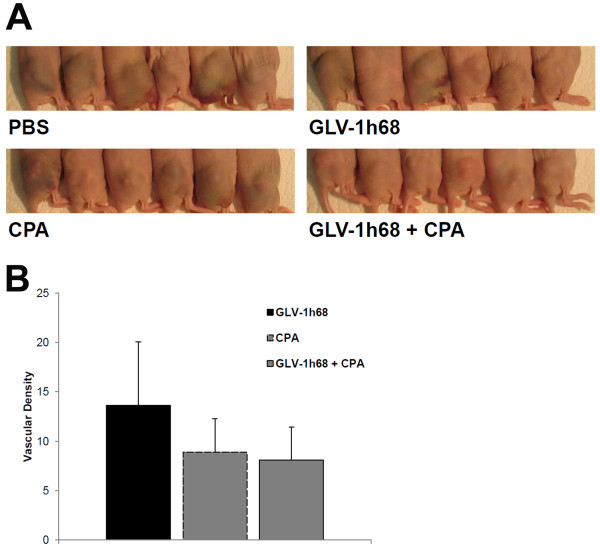
**Phenotypes of s.c. implanted PC14PE6-RFP tumors and number of CD31-positive blood vessels. (A)** Photographic images of subcutaneously implanted PC14PE6-RFP tumors were taken 7 days post PBS-, GLV-1h68-, CPA- or combination treatment begin. **(B)** Vascular density was measured in CD31-labelled cross-sections of GLV-1h68-, CPA- or combination treated tumors 7 days after treatment start. Shown are the mean values +/- s.d.

We further could observe that especially untreated subcutaneously implanted PC14PE6-RFP tumors but also GLV-1h68- or CPA-treated tumors had a hemorrhagic phenotype 7 dpi (Figure 5A), in striking contrast to tumors after combination treatment, which appeared either white or skin-colored. Notably, at the time point of infection, tumors of the combination group have already been slightly blue demonstrating that by treatment with GLV-1h68 in combination with CPA, tumors lose their hemorrhagic phenotype. We finally analyzed the vascular density by CD31 staining of tumor sections 7 dpi. Indeed, there was a decrease in the number of blood vessels after CPA and combination treatment compared to GLV-1h68-treated tumors (Figure 5B), albeit this was not significant.

## Discussion

The efficiency of the oncolytic VACV GLV-1h68 in treating different human or canine cancers in mice has been demonstrated in several preclinical studies [12-28]. Moreover, currently two clinical trials with GLV-1h68 as intervention are carried out (http://www.clinicaltrials.gov). One great advantage of oncolytic viruses for the treatment of cancers comprises their capability to specifically kill cancerous cells whereas normal cells are not affected. In the current study, we investigated the effect of a combined treatment strategy consisting of oncolytic VACV GLV-1h68 and the alkylating agent CPA on the growth of human lung adenocarcinoma PC14PE6 xenografts. Synergistic effects of diverse oncolytic viruses and CPA have been described in the literature [31-41]. Suggested mechanisms are, if specified, mostly due to the immuno-modulatory properties of CPA. CPA is known to suppress the host innate immunity and inhibition of intratumoral infiltration of mononuclear cells has been described, thus probably extending the survival of the virus within the tumors [33,36,37]. When used in a low- dose metronomic (LDM) schedule, CPA can also, exert antiangiogenic effects [43-45].This seems to be based on the induction of thrombospondin-1 which in turn probably acts antiangiogenic through binding to CD36 [46] or through binding and sequestering VEGF [47].

In our study, we could show that combination therapy with GLV-1h68 and CPA led to a significant growth inhibition of PC14PE6-RFP xenografts compared to GLV-1h68 alone from day 21 post injection on (Figure 3B). Analysis of the viral load of combination-treated or GLV-1h68-treated tumors revealed that the better therapeutic effect was not due to an enhanced viral titer in the tumors. Moreover, after combination treatment we did not see enhanced viral titers in the organs of mice suggesting that CPA has no immunosuppressive effect in our experimental model. Immune-related effects of the investigated therapy were not explored in detail in the present study. However, preliminary data obtained by histological analysis of CPA- and non-CPA-treated tumors 7 dpi revealed no significant difference in the content of CD68-positive immune cells, e.g. monocytes/macrophages (Additional file 1). Furthermore depletion of phagocytic cells especially monocytes and macrophages by clodronate liposomes did result in lesser tumor growth retardation than by treatment with CPA alone (Additional file 2). Both observations are again hinting that CPA has no effect on cells of monocytic origin in the PC14PE6-RFP model. By protein profiling of lysates of untreated, CPA-, GLV-1h68- and combination treated tumors, we found the expression of several pro-inflammatory cytokines and chemokines of host origin to be up-regulated in tumor tissues after virus infection and even after combination treatment, although to a lesser extent (Table 3). The majority of these cytokines/chemokines and pro-inflammatory proteins, like eotaxin, MIP-1 beta, MCP-1, MCP-3, MCP-5, TNF-alpha, and MPO are known to be produced by activated macrophages in the inflamed tissue. Similar results also have been obtained in xenograft models of human pancreatic [18], prostate [22] and hepatocellular [26] as well as canine mammary carcinomas [28] suggesting that it is a general mechanism of GLV-1h68 to induce strong innate host immune responses in tumors of different origins. It is also very interesting to note that after virus infection or combination treatment the CCR5 ligands MIP-1 beta/MIP-3 beta or RANTES, respectively, are overexpressed. The CCR5 ligand chemokine pathway is, among other molecular pathways, known to be consistently activated during immune-mediated cancer rejection as well as other immune-mediated tissue destruction processes, as described in the “Immunologic Constant of Rejection (ICR)” [48]. The cytokine Oncostatin-M, which is up-regulated after VACV infection, belongs to the IL-6 family and has cytostatic activities on a number of tumor cell lines [49-51]. The only protein that was found to be down-regulated upon viral infection was EGF. Interestingly, mutations of the EGF receptor (EGFR) have been associated with several types of cancers, including lung cancer, and targeted therapy using small molecules disrupting kinase activity of EGFR results in considerable clinical benefit in lung cancer patients [4]. Moreover, Goswami *et al.*[52] described a paracrine loop between breast carcinoma cells (producing CSF) and macrophages (producing EGF) leading to increased carcinoma cell invasion and they suggested that disabling this loop might result in clinical benefit in the treatment of cancer. So, it is remarkable that by virotherapy with Vaccinia virus endogenous EGF levels are reduced possibly resulting in inhibition of downstream pro-survival signaling pathways or inhibition of tumor cell invasion.

Combination treatment of GLV-1h68 and CPA leads to significant up-regulation of M-CSF-1, MCP-1 and MCP-5, which have pro-inflammatory activities (Table 3B) and again to down-regulation of EGF compared to untreated controls. Concentrations of Apo A-I, VCAM-1, Fibrinogen and vWF are found to be decreased after combination treatment. Apo A-I has anti-inflammatory [53,54] but also anti-thrombotic [55] activities. VCAM-1 is expressed by the cytokine-activated endothelium and mediates leukocyte-endothelial cell adhesion. It is also known that in several types of cancers VCAM-1 is aberrantly expressed on the surface of tumor cells thereby tethering macrophages to tumor cells and generating favorable conditions for tumor angiogenesis, invasion and metastasis [56,57]. Recently, a study reported about an EGF-enhanced VCAM-1 expression promoting macrophage and glioblastoma cell interaction and tumor cell invasion [57]. In another work a VCAM-1-mediated tumor immune evasion has been described [58,59]. The authors found that overexpression of VCAM-1 by cancer cells led to decreased apoptosis of tumor cells and a significant decrease in the number of tumor-infiltrating CD8+ T cells expressing VCAM-1. Therefore, reduction of VCAM-1 and EGF by combination treatment might have favorable effects in terms of inhibition of tumor cell invasion and tumor immune evasion. Fibrinogen plays a pivotal role in the coagulation cascade. vWF is synthesized by endothelial cells and megakaryocytes and promotes the adhesion of platelets to sites of vascular injury. Interestingly, the latter four factors, Apo A-I, VCAM-1, Fibrinogen and vWF, which are found to be decreased after combination treatment, are either expressed by endothelial cells or are present in the blood, hinting that combination therapy has an effect on the tumor vasculature in the PC14PE6-RFP model. Compared to CPA treatment alone, infection with GLV-1h68 or combination treatment leads to an up-regulation of several pro-inflammatory cytokines and chemokines (Table 3C and D), like seen in the virus-treated versus untreated tumors, affirming that virus infection leads to a pro-inflammatory status within the tumor. Moreover, compared to monotherapy with CPA, lower levels of VCAM-1 are observed upon combination treatment. Interestingly, after CPA or combination therapy the only protein that was found to be significanlty decreased compared to respective controls was vWF (Table 3E and F). This indicates that the primary effect of CPA in this model is caused by an effect on the vasculature and not by alteration of the immune response. However, it is speculating to assume if the lower levels of these factors are due to a down-regulation on the level of protein expression or are a result of the lower density of blood vessels found in tumors after CPA or combination treatment.

Taken together, these data show that pro-inflammatory cytokines and chemokines are elevated in PC14PE6-RFP tumors of GLV-1h68-treated mice (Table 3A, B, C, and D). But more importantly, levels of factors either expressed by endothelial cells or present in the blood are found to be decreased after CPA or combination treatment (Table 3B, D, E, and F). We therefore suppose that in the PC14PE6-RFP model the enhanced tumor growth inhibition observed after combination therapy is due to an effect on the vasculature rather than an immunosuppressive action of CPA. However, it is conceivable that the modulation of the vasculature may alter the following immunological response to VACV e.g. by hampering immune recruitment to the tumor site.

Especially untreated PC14PE6-RFP tumors, but also GLV-1h68 or CPA-treated tumors have a hemorrhagic phenotype (Figure 5A). This seems to be a hallmark of the PC14PE6 cell line since Yano *et al.* already described the development of bloody pleural effusions after *i.v.* injection of PC14PE6 cells [60]. Only recently, Weibel *et al.* showed that after subcutaneous implantation of PC14PE6 cells malignant effusion concomitant hematoma formation occurs at the tumor site, and, during oncolytic virotherapy using GLV-1h68 and especially its derivative GLV-1h108, which encodes a single chain antibody against VEGF, tumor-associated hematoma disappeared [61]. Remarkably, in the present study we could show that after combination treatment PC14PE6-RFP tumors lose their hemorrhagic phenotype. Since at the time point of treatment start, tumors of the combination group already showed a slight blue color, CPA treatment seems not only to prevent the development of the hematoma but to actively rebuild a non-hemorrhagic appearance when used in combination with oncolytic Vaccinia virus GLV-1h68. This additionally indicates that in the PC14PE6-RFP xenograft model combination therapy with GLV-1h68 and CPA acts on the tumor vasculature. To further support this hypothesis, we analyzed the vascular density in CD31-labelled tumor cross-sections 7 dpi. Indeed, there was a decrease in the number of blood vessels after CPA and combination treatment compared to GLV-1h68-treated tumors (Figure 5B), albeit this was not significant. The presence of CD31-positive blood vessels after CPA treatment, further suggest that CPA seems to have no immunosuppressive effect, since CD31 is involved in leukocyte trafficking to sites of inflammation. Previous work in our laboratory demonstrated that GLV-1h68 does not destroy endothelial cells in tumors and that the tumor vasculature in infected tumors is still functional [62]. In the matter of the significance of tumor vasculature in tumor progression, therapeutic approaches additionally targeting the tumor endothelium may contribute to a better therapeutic outcome. Therefore, combination strategies composed of an agent, which directly kills tumor cells on the one hand (in our case GLV-1h68), and an additional therapeutics, which targets normal cells within the tumors, e.g. tumor vasculature, are particularly advantageous. In this study, such a combination therapy consisting of oncolytic VACV GLV-1h68, destroying tumor cells, and CPA, presumably targeting tumor vasculature, could successfully be established for the PC14PE6-RFP tumor model.

## Conclusions

Our data demonstrate that combination treatment of GLV-1h68 and CPA results in strongly enhanced antitumor efficacy in the PC14PE6-RFP tumor model. Results presented here suggest that improved tumor control achieved by combining GLV-1h68 with CPA is due to the action of CPA on the tumor vasculature. Yet, the effect of CPA may be cancer cell specific as well as highly dose dependent and may also be mouse model specific. Nevertheless, these results justify the design of additional preclinical studies in other highly angiogenic tumor models. Moreover, the data shown here demonstrate that CPA as a chemotherapeutic agent can also be combined successfully with GLV-1h68 strain (GL-ONC1), which is currently evaluated in phase I/II clinical trials as monotherapy and could be considered as combination therapy in human clinical trials.

## Abbreviations

CPA: Cyclophosphamide; FBS: Fetal bovine serum; GFP: Green fluorescent protein; Hpi: Hours post infection; LIVP: (Lister strain from the Institute of Viral preparations, Moscow, Russia); MOI: Multiplicity of infection; MTT: 3-(4,5-dimethylthiazol-2-yl)-2,5-diphenyltetrazolium bromide; Pfu: Plaque forming units; RFP: Red fluorescent protein; RUC: Renilla luciferase; s.d.: Standard deviation; VACV: Vaccinia virus.

## Competing interests

EH and SW are recipients of postdoctoral fellowships of the University of Würzburg from a research service grant provided by Genelux Corporation. AAS is salaried employee of Genelux Corporation and has personal financial interests in Genelux Corporation. The funders had no role in study design, data collection and analysis or decision to publish. This work was supported by a research grant and a service grant from Genelux Corporation (R&D facility in San Diego, CA, USA).

## Authors’ contributions

EH and SW designed the study, performed the experiments, statistical analyses and interpretation of data and wrote the manuscript. AAS participated in conceiving the study and writing the manuscript. All authors read and approved the final manuscript.

## Supplementary Material

Additional file 1 **Histological analysis of CD68-positive immune cells in PC14PE6-RFP tumors.** PC14PE6-RFP tumor-bearing mice were either i.v. injected with 1×10^7^ pfu of GLV-1h68 or PBS as control and/or received i.p. injections with 140 mg/kg bodyweight CPA at day 0 and with 100 mg/kg bodyweight at days 1, 3 and 7. Tumors were harvested 7 dpi. **(A)** Whole tumor cross-sections of PBS-, CPA-, GLV1h68- or combination-treated tumors were labelled with an anti-rat CD68 antibody (Abcam, Cambridge, UK) and a secondary DyLight 649-conjugated donkey anti-rat antibody (Dianova, Hamburg, Germany) to visualize the presence of CD68-positive leukocytes. All confocal images are representative examples for respective groups. **(B)** Mean fluorescence intensity of CD68-labelling was determined using Image J software (http://rsbweb.nih.gov/ij). For all experiments the mean value was calculated for 12 images (four images of three different PBS-, CPA-, GLV-1h68- or combination-treated tumors) and presented as mean values with standard deviations. Click here for file

Additional file 2 **Depletion of phagocytic cells by clodronate liposomes.** PC14PE6-RFP tumor-bearing mice were either i.v. injected with 1×10^7^ pfu of GLV-1h68 or PBS as control or received i.p. injections with 140 mg/kg bodyweight CPA at day 0 and with 100 mg/kg bodyweight at days 1, 3 and 7 or i.p. injections with 200 μl Clodronate liposomes (CLIP) at days 0, 1, 3 and 7 post treatment start. Clodronate liposomes were obtained from clodronateliposomes.com (N. van Rooijen, Amsterdam, The Netherlands) at a concentration of 7 mg/ml. Tumor growth was monitored for 14 days. Click here for file
