# Supplementary figures and images for: Combination treatment with oncolytic Vaccinia virus and cyclophosphamide results in synergistic antitumor effects in human lung adenocarcinoma bearing mice
Source: J Transl Med. 2014 Jul 17;12:197. doi: 10.1186/1479-5876-12-197 (PMC4105246; doi:10.1186/1479-5876-12-197)

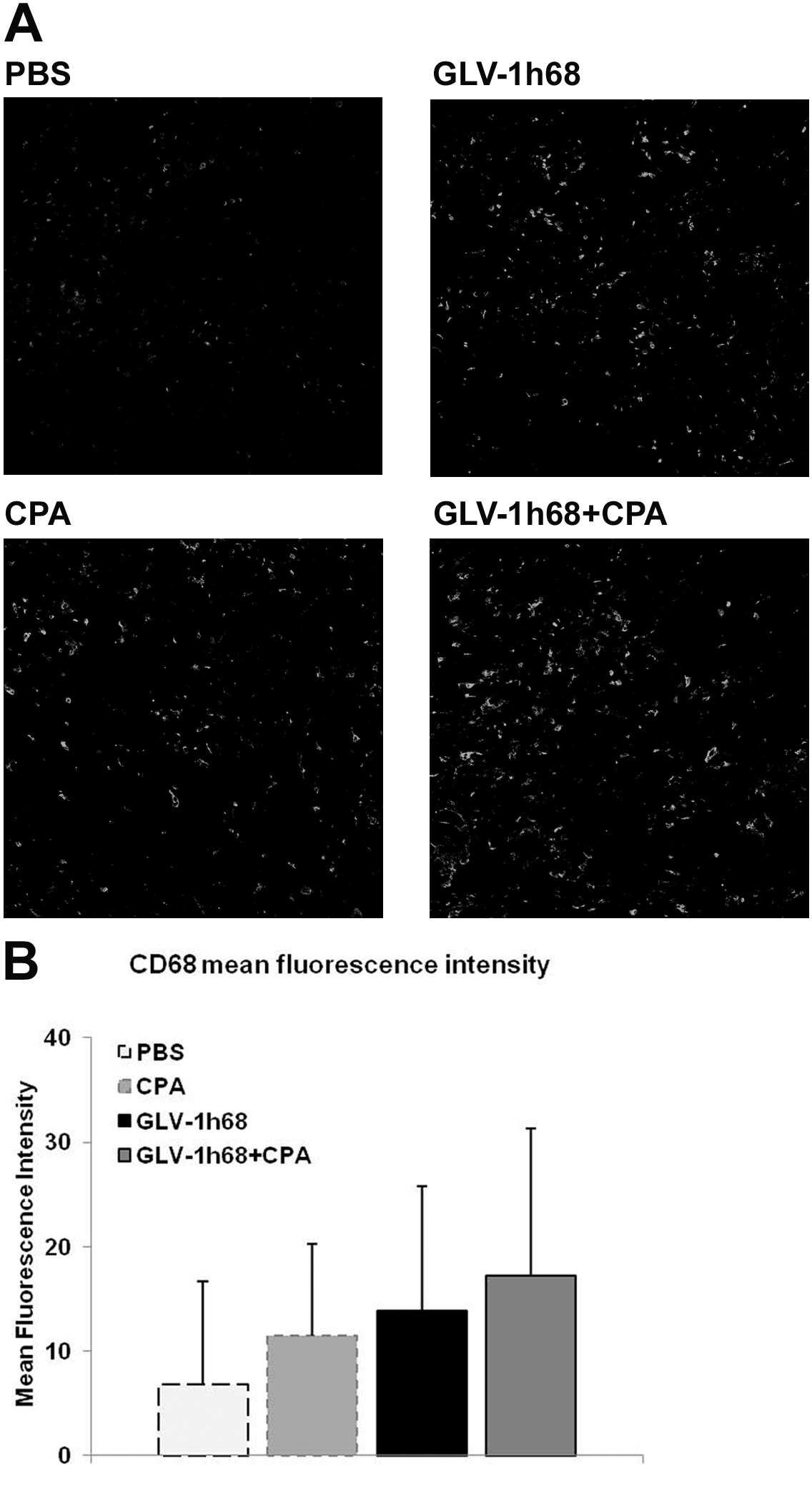

Supplement: Additional file 1 — Histological analysis of CD68-positive immune cells in PC14PE6-RFP tumors. PC14PE6-RFP tumor-bearing mice were either i.v. injected with 1×10^7^ pfu of GLV-1h68 or PBS as control and/or received i.p. injections with 140 mg/kg bodyweight CPA at day 0 and with 100 mg/kg bodyweight at days 1, 3 and 7. Tumors were harvested 7 dpi. (A) Whole tumor cross-sections of PBS-, CPA-, GLV1h68- or combination-treated tumors were labelled with an anti-rat CD68 antibody (Abcam, Cambridge, UK) and a secondary DyLight 649-conjugated donkey anti-rat antibody (Dianova, Hamburg, Germany) to visualize the presence of CD68-positive leukocytes. All confocal images are representative examples for respective groups. (B) Mean fluorescence intensity of CD68-labelling was determined using Image J software (http://rsbweb.nih.gov/ij). For all experiments the mean value was calculated for 12 images (four images of three different PBS-, CPA-, GLV-1h68- or combination-treated tumors) and presented as mean values with standard deviations. [file 1479-5876-12-197-S1.tiff]

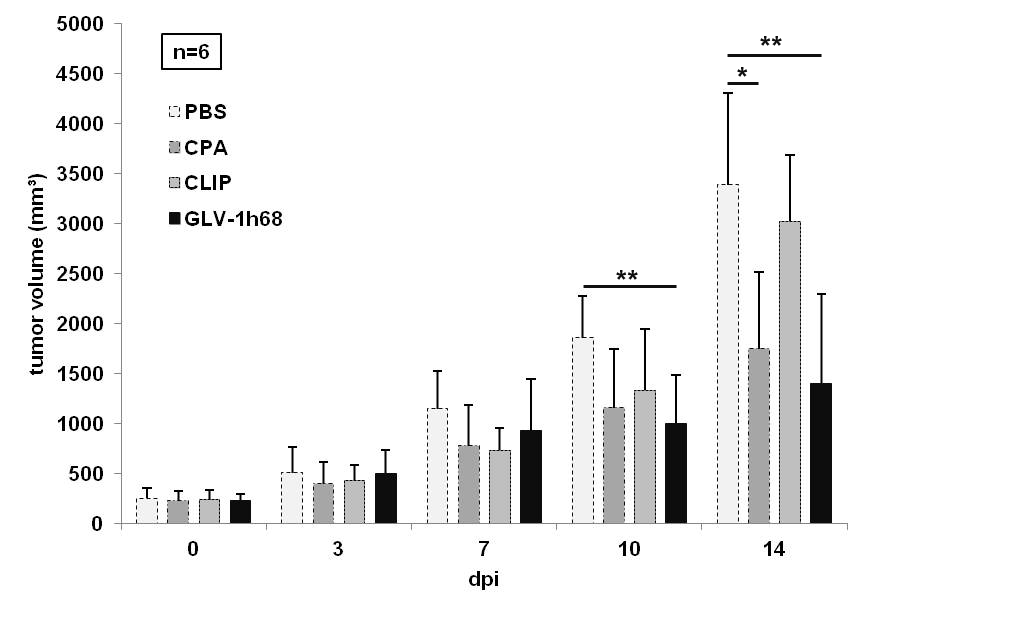

Supplement: Additional file 2 — Depletion of phagocytic cells by clodronate liposomes. PC14PE6-RFP tumor-bearing mice were either i.v. injected with 1×10^7^ pfu of GLV-1h68 or PBS as control or received i.p. injections with 140 mg/kg bodyweight CPA at day 0 and with 100 mg/kg bodyweight at days 1, 3 and 7 or i.p. injections with 200 μl Clodronate liposomes (CLIP) at days 0, 1, 3 and 7 post treatment start. Clodronate liposomes were obtained from clodronateliposomes.com (N. van Rooijen, Amsterdam, The Netherlands) at a concentration of 7 mg/ml. Tumor growth was monitored for 14 days. [file 1479-5876-12-197-S2.tiff]
